# Immunopathological immunohistochemical study of low pathogenic avian influenza virus H5N1 infection in lovebirds (*Agapornis* spp.) in Indonesia

**DOI:** 10.14202/vetworld.2019.1472-1477

**Published:** 2019-09

**Authors:** Zulfikhar Zulfikhar, Raden Wasito, Hastari Wuryastuti

**Affiliations:** 1 Doctoral Study Program, Veterinary Science, Faculty of Veterinary Medicine, Gadjah Mada University, Yogyakarta, Indonesia; 2 Department of Pathology, Faculty of Veterinary Medicine, Gadjah Mada University, Yogyakarta, Indonesia; 3 Department of Internal Medicine, Faculty of Veterinary Medicine, Gadjah Mada University, Yogyakarta, Indonesia

**Keywords:** avian influenza virus H5N1, immunohistochemistry, Indonesia, lovebird, lung, streptavidin-biotin

## Abstract

**Background and Aim:**

To date, persistent infection with low pathogenic avian influenza virus (AIV) subtype H5N1 (LPAI H5N1) in chickens is widespread in Indonesia. Commercially available ornamental birds on markets play an important role in triggering the occurrence of AIV outbreaks. Thus, the presence of AIV in ornamental birds such as lovebirds needs to be closely monitored. Here, the presence of LPAI H5N1 was investigated in lovebirds with no clinical signs that, although apparently healthy, can be a continuous source of AIV transmission to other sensitive birds such as chickens and even humans.

**Materials and Methods:**

In the present study, 30 lovebirds were necropsied. The immunopathology of the lungs, which had hemorrhages, was immunohistochemically studied using the streptavidin-biotin (SB) method to stain for LPAI H5N1. Polyclonal antibodies against the nucleoprotein or monoclonal antibodies against the hemagglutinin of the AIV subtype H5N1 were used to identify these antigens in lung tissue samples.

**Results:**

LPAI H5N1 was detected in the lungs of all lovebirds according to the brownish colored deposits in the histological samples. The highest intensity of the SB staining was found in the lumina of vascular capillaries and the cell membranes of air capillaries. The discovery of LPAI H5N1 in lovebirds increases the environmental health risk and raises the possibility of exposure to AIV. Thus, it is very important to improve the mechanisms that control the traffic of ornamental birds between regions and islands, as well as the AIV vaccination strategies related to the prevention, control, and eradication of AIV in Indonesia, and these procedures should be reevaluated.

**Conclusion:**

The present study proves that lovebirds are infected with LPAI subtype H5N1. Even if ornamental birds such as lovebirds do not show any clinical signs, they are likely to be a persistently infected with LPAI H5N1. Therefore, ornamental birds might be a continuous source of LPAI H5N1 infections in other sensitive birds, including poultry, and may also be responsible for virus transmission to humans.

## Introduction

The avian influenza virus subtype H5N1 (AIV H5N1) has been prevalent in poultry, including ornamental birds, such as lovebird [1] in Indonesia since 2003 and the most frequent outbreaks of AIV infections in these birds have been in Java [2]. Lovebirds usually not vaccinated. They are commonly found in bird markets and communities, making them a potential source of AIV transmission to poultry and even humans. The AIV subtype H5N1 often has a low virulence level [3]. Birds infected by this subtype are usually subclinical, meaning that infected birds do not show any clinical signs of illness, and appear to be healthy, are a continuous source of AIV infections in more sensitive birds [4,5].

Detection of the AIV subtype H5N1 in ornamental birds is very important to identify new AIV subtypes that are likely to cause AIV epidemics in poultry [6]. This is possible because AIV H5N1 subtypes are prone to antigenic drift [7]. Humans can also be affected through direct contact with infected birds or contaminated materials [8,9], because AIV also has the ability to cross-react after genetic reassortment [10]. Cases of AIV transmission in humans have been reported in nine Indonesian Provinces, namely, DKI Jakarta, Banten, West Java, Central Java, East Java, North Sumatra, West Sumatra, Lampung, and South Sulawesi [11]. Live poultry in markets, including chickens and commercial ornamental birds, plays an important role in triggering the occurrence of AIV outbreaks [12]; thus, the present of AIV subtypes in poultry needs to be closely monitored requires serious attention [7,10,13]. Diagnostic tools are very important to eradicate this disease by monitoring its occurrence and preventing disease outbreaks [14]. To date, the confirmation of an AIV diagnosis is still based on the isolation and identification of the virus with testing standards, such as virus isolation, that are time consuming [15,16], whereas reverse transcriptase-polymerase reactions (RT-PCRs) require strictly regulated facility, namely, Biosafety Level II or III laboratories and expensive molecular biology reagents. Virus isolation and RT-PCRs can result in contamination of the laboratory and the environment which may further spread AIV infections in poultry and humans [17-19].

It is very important to develop environmentally friendly, fast, inexpensive, and accurate early diagnosis techniques [12,20] to minimize the incidence of AIV outbreaks in Indonesia. In the present study, we describe an immunopathological approach based on immunohistochemistry to detect AIV infections. We suggest that this biotechnological method fulfills the aforementioned criteria and should be applied to control and reduced zoonotic risks, such as bird flu pandemics in Indonesia [21-25].

Here, the presence of low pathogenic avian influenza (LPAI) H5N1 was investigated in lovebirds with no clinical signs that, although apparently healthy, can be a continuous source of AIV transmission to other sensitive birds, such as chickens and even humans.

## Materials and Methods

### Ethical approval

This study only worked on samples obtained from the field, so, it does not need ethical approval.

### Lovebirds samples

Thirty lovebirds were collected from several commercial ornamental birds’ keepers in an endemic area of avian influenza in West Java, Indonesia. All lovebirds were necropsied, and the AIV target organ, the lungs [26] were macroscopically examined for pathological changes. The lungs were then collected and fixed for 24 h with 10% neutral buffered formalin for further histopathological processing, including routine stainings with hematoxylin-eosin. Moreover, streptavidin-biotin (SB)-based immunohistochemistry was conducted using antibodies against the nucleoprotein and hemagglutinin (HA) antigens of the AIV subtype H5N1.

### Lung preparation for histopathology and immunohistochemistry

Before the histopathological hematoxylin-eosin and immunohistochemical SB (IHC SB), stainings were carried out; the formalin-fixed lungs were processed with automatic histotechnician. The lungs were dehydrated in increasing concentrations of ethanol solutions (80% for 2 h, 95% for 2 h, 95% for 1 h, and 100% 3× for 1 h each), washed with xylene (3× for 1 h each), and soaked in liquid paraffin 3× for 2 h each. After dehydration, lung tissue samples were soaked in dilute paraffin solution and cut into 3-5 µm thick slices using a microtome. These lung samples were used for the histopathological examination with routine hematoxylin-eosin stainings and IHC approaches using antibodies directed against nucleoprotein and HA of the AIV subtype H5N1.

### Histopathological staining

For the routine hematoxylin-eosin staining, the lung tissue preparations of 3-5 µm thickness were deparaffinized with xylene (3× for 5 min each) followed by decreasing ethanol concentrations (absolute, 95% and 50% for 5 min each). The slices were washed with distilled water and phosphate-buffered saline (PBS 2× for 1 min each) before staining them with Harris hematoxylin for 20 min. Afterward, they were washed with distilled water, dyed in acid alcohol 2-3 times, washed again with distilled water for 1 min and another 15 min, and dipped into eosin solution for 2 min. Finally, the samples were placed into ethanol (96%, absolute, both 2× 3 min each), and washed with xylene (2× for 5 min each). The histopathological lung sections were covered with a coverslip using glycerol for subsequent examinations with a microscope.

### IHC stainings against AIV antigen

The lungs tissue samples were deparaffinized with xylene (3× for 5 min each), rehydrated with decreasing ethanol concentrations (100%, 95%, and 50%, for 3 min each), washed with distilled water, and finally cleaned with 0.05M PBS, pH 7.1. The tissue slices were then immersed into 3% H_2_O_2_ solution in absolute methanol to inactivate endogenous peroxidase activity, washed with PBS 3× and soaked in normal rat serum for 20 min. The slice preparations were incubated afterward with polyclonal anti-nucleoprotein AIV H5N1 or monoclonal anti-HA AIV H5N1 antibodies (Santa Cruz Biotechnology) for 45 min at 20°C. The tissue preparations were then washed with sterile distilled water and immediately incubated with secondary biotin-labeled goat anti-rabbit IgG antibodies (Dako) for 10 min. Next, the samples were conjugated with SB-horseradish peroxidase (BD PharMingen, San Diego, CA) for 10 min at 20°C before 3,3-diaminobenzidine solution (Zymed Corp., San Francisco, CA) was applied for 3 min for antibody detection. The slice preparations were also given basic coloring hematoxylin, washed with distilled water, dehydrated, and sealed with a coverslip and glycerol for subsequent observations under a microscope.

The IHC SB stainings for nucleoprotein and HA antigens of the AIV subtype H5N1 were analyzed descriptively based on color changes, namely, the presence of a brownish color representing the antigen deposition in the lung tissues.

## Results

**Figure-1:**
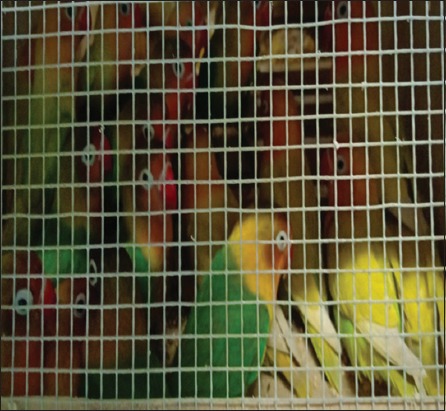
A lovebird used in the present study without a clinical sign.

**Figure-2:**
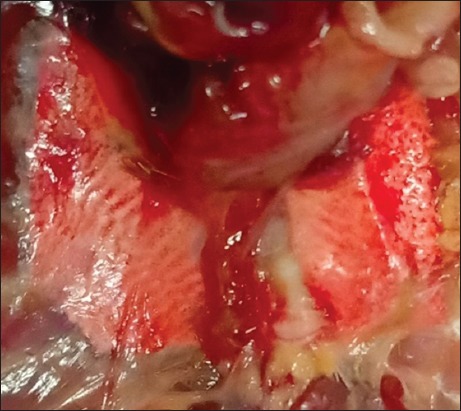
Lung of a lovebird. Notice the swollen, severe, and diffuse spotted and/or linear congestion and hemorrhages in the lung.

**Figure-3:**
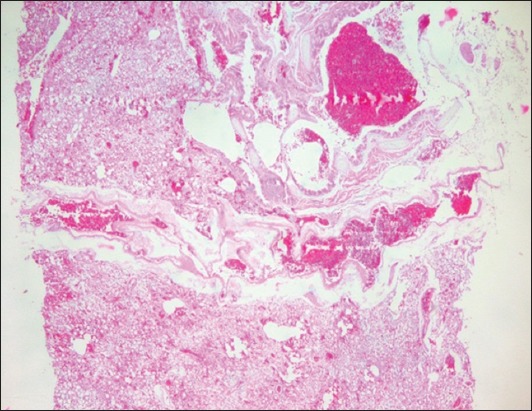
Lung histopathology in a lovebird. Notice the severe and diffuse congestion and hemorrhages in the lung (hematoxylin-eosin staining, 250×).

**Figure-4:**
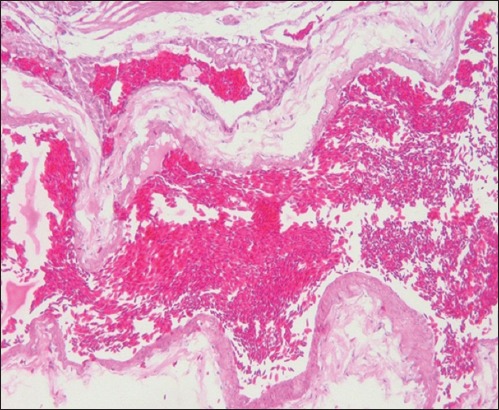
Histopathology a higher magnification. Notice the severe and diffuse congestion and hemorrhages in the lung (hematoxylin-eosin staining, 500×).

**Figure-5:**
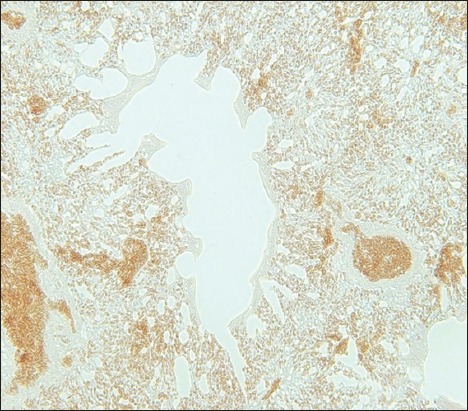
The lung of a lovebird is positive for the avian influenza virus nucleoprotein displayed as a brownish-colored deposit in the lumen of a vascular capillary and the cell membrane of an air capillary (streptavidin-biotin staining, 500×).

**Figure-6:**
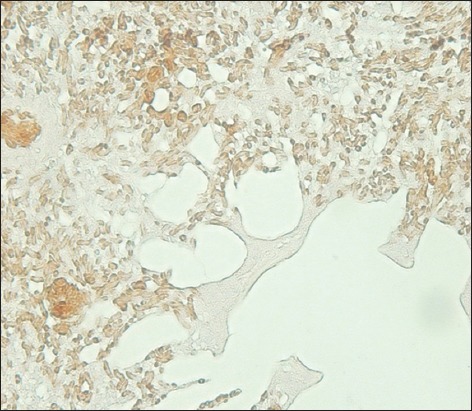
The lung tissue of a lovebird at a higher magnification. The sample is positive for the avian influenza virus nucleoprotein according to a brownish deposit in the lumen of a vascular capillary and the cell membrane of an air capillary (streptavidin-biotin staining, 1000×).

In the present study, none of the 30 lovebirds collected from local ornamental bird farms in the West Java region, Indonesia, did present any clinical signs of AIV infection. They appeared to be healthy (Figure-1). However, all examined birds had in the necropsy macroscopic lung lesions. The lungs appeared swollen, were opaque and exhibited severe and diffuse congestion and hemorrhages (Figure-2). Histopathological lesions such as severe and diffuse congestion and hemorrhages were also seen in all sampled lungs (Figures-3 and 4). Moreover, immunopathological changes were identified IHC SB with polyclonal antibodies directed against the nucleoprotein (NP poAb) of AIV H5N1 indicated that all lovebirds were positive for AIV infection in the target organ, the lungs [24]. In the IHC SB, the lungs infected with AIV showed deposited NP antigen in the form of brownish-colored precipitates. The NP poAb precipitates were mainly visible in the lumina of vascular capillaries and the cell membranes of air capillaries (Figures-5 and 6).

**Figure-7:**
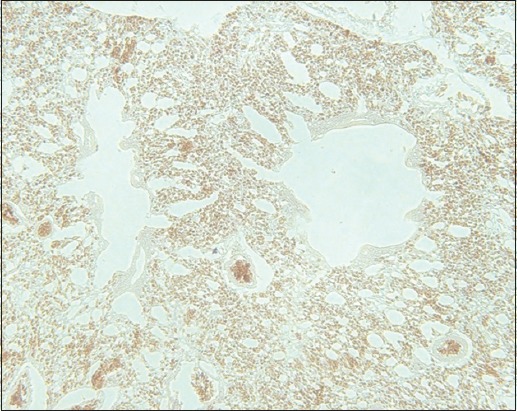
The lung of lovebird stains positive for the avian influenza virus hemagglutinin of the subtype H5N1 shown as a brownish deposit in the lumen of a vascular capillary and the cell membrane of an air capillary (streptavidin-biotin staining, 500×).

**Figure-8:**
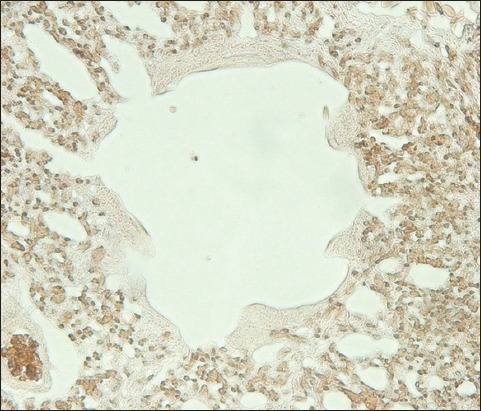
Lung histopathology of a lovebird at a higher magnification. It is positive for the avian influenza virus hemagglutinin of subtype H5N1 presenting as a brownish-colored deposit in the lumen of a vascular capillary and the cell membrane of an air capillary (streptavidin-biotin staining, 1000×).

Furthermore, it was determined whether the AIV was a specific subtype of H5N1 using IHC SB with a monoclonal antibody against HA (HA moAb) AIV subtype H5N1. In this IHC SB, the lungs were also positive for that specific HA moAb, and the precipitates were again mainly deposited in the lumina of vesicle capillaries and air capillary cell membranes (Figures-7 and 8).

## Discussion

Avian influenza is an infectious disease in birds caused by the influenza virus type A. This virus is an RNA virus that belongs to the *Orthomyxoviridae* family and is classified based on the serological subtype of the surface glycoproteins, HA, and neuraminidase (NA). To date, poultry has been exposed to 16 HA (H1-H16) and 9 NA (N1-9) subtypes in different combinations [27]. Based on the molecular identity of HA proteins and their ability to cause outbreaks in chickens, the influenza virus is also grouped into highly pathogenic avian influenza (HPAI) and LPAI. The AIV subtypes H5 and H7 can mutate from the LPAI form to HPAI through antigenic drift [7]. In addition, the H5 and H7 subtypes can also induce cross-reactions in hosts, thus allowing the formation of new AIV strains that are capable of being transmitted to humans [10].

The AIV is widespread in the world and has infected various kinds of birds. In poultry species, the orders *Anseriformes* (water birds) and *Charadriiformes* (shorebirds and gulls) are natural hosts of the AIV [28]. Since 2003, outbreaks of the highly pathogenic AIV subtype H5 (HPAI H5) have occurred in Asia in wild birds and poultry, and these outbreaks spread subsequently to Europe, Africa, and North America. The HPAI H5N1 epidemic has also affected Indonesia [1,2]. West Java, East Java, and Central Java are reported as the main places that are consistent sources of AIV outbreaks in poultry. Java is listed as the region in Indonesia with the highest number of AIV cases in birds [2].

The low pathogenic AIV H5N1 (LPAI H5N1) has been detected in Indonesia [23,26]. In general, the AIV subtype H5N1 has low virulence [3], and birds infected with this subtype are asymptomatic, meaning that infected birds do not show any clinical signs of illness, and appear to be healthy, but are a continuous source of AIV infections in other more sensitive birds [4,5].

The primary organ target of the AIV is lung [26]. In the present study, the lungs appeared swollen and opaque and had severe, diffuse, spotted and linear congestion, and hemorrhages. It was reported that AIV infections in chickens are characterized by lesions in the lungs as observed in the present study [23,25]. Chickens infected with AIV also show significant hemorrhagic lesions [29]. Based on the virus pathogenesis, AIV is thought to cause viremia in the host body in the same way as other endotheliotropic viruses. Activation of polymorphonuclear and mononuclear inflammatory cells, as well as systemic cytokine release, results in a predisposition to organ lesions [29].

Based on the results of the IHC SB tests using NP poAb and HA moAb, lovebirds were infected with the LPAI H5N1. LPAI H5N1 virions were detected in epithelial cells of the parabronchi and secondary bronchi as brownish deposits indicative of IHC antibody-antigen reactions. The deposits of LPAI H5N1 particles were in the cytoplasm and the nucleus. In the present study, we examined the lungs because they are the primary target organs of the AIV [26]. It has been reported that wild birds infected with the LPAI subtype H5N1 did not show clinical signs [30].

Every individual or poultry farmer, including ornamental birds farmers, must continuously monitor their poultry or birds for any possible clinical signs. In the field, the clinical signs of LPAI subtype H5N1, in general, are difficult to distinguish from other viral infections, such as Newcastle disease virus (NDV). The clinical symptoms of LPAI subtype H5N1 are similar to NDV, among others: The head appears to move irregularly to the left, right, and down (torticollis), claws appear curled toe paralysis, respiratory problems, and acute diarrhea [1].

Thus, the presence of the LPAI subtype H5N1 in ornamental birds, especially in lovebirds originating from local farms in West Java could result in an increased risk of exposure to LPAI subtype H5N1 infections in other birds, including poultry. The results of this study regarding LPAI infections are important because of the nature of the H5N1 subtype which is prone to antigenic drift [7] and antigenic shift (genetic reassortment) [10]. Therefore, increased vigilance is required because there is the possibility of an HPAI outbreak in poultry in Indonesia. The HPAI subtype H5N1 can cause significant mortality in infected chickens due to the involvement of almost all pulmonary lobes. HPAI infection in poultry causes significant pathological lesions in the form of congestion, hemorrhages, and edema [31]. Attention should also be given to the possible emergence of a new type A influenza virus pandemic in Indonesia.

Outbreaks in birds infected with the LPAI subtype H5N1 will reduce the efficiency of programs aiming at the control and eradication of the AIV. For the efficient control of AIV transmission and infection, the efficacy of vaccines and vaccination programs need to be improved not only by biotechnological advances but also by a comprehensive control strategy, including strict biosurveillance programs, better education of bird breeders, rapid, economic environmentally friendly and accurate diagnosis, and safe disposal of AIV-infected poultry [31].

## Conclusion

The present study demonstrated that lovebirds were infected with the LPAI subtype H5N1. The lovebirds did not show any clinical signs, but they were likely persistently infected with the LPAI subtype H5N1. These birds might become a continuous source of LPAI subtype H5N1 infections in other sensitive birds, including poultry and be responsible for virus transmission to humans.

## Authors’ Contributions

ZZ carried out the collection of lovebird in West Java and performed streptavidin-biotin staining. RW prepared the lungs for the histopathological streptavidin-biotin stainings. HW played a role in performing the streptavidin-biotin stainings and interpreting the results. All authors discussed the data before giving their consent to publish the manuscript.
